# Gender disparities in multiple myeloma publications

**DOI:** 10.1002/jha2.470

**Published:** 2022-06-15

**Authors:** Aala Dweik, Hadeel Dweik, Hira Mian, Meera Mohan, Carolina Schinke, Samer Al Hadidi

**Affiliations:** ^1^ Department of Medicine, Faculty of Medicine University of Jordan Amman Jordan; ^2^ Department of Oncology McMaster University Hamilton Canada; ^3^ Department of Internal Medicine Division of Hematology and Oncology Medical College of Wisconsin Milwaukee Wisconsin USA; ^4^ Department of Hematology and Oncology, Myeloma Center Winthrop P. Rockefeller Cancer Institute University of Arkansas for Medical Sciences Little Rock Arkansas USA

**Keywords:** academia, disparities, gender, Multiple myeloma, publications

## Abstract

Gender disparities exist in academia and are disproportionately affecting females. We conducted a cross‐sectional study to analyze gender disparities in multiple myeloma (MM) publications. A total of 679 publications with 8898 authorships were analyzed. The mean number of authors for females vs. males, per publication, was 4.4 and 8.7, respectively. Females constituted a third of the total authors. Female first authors, corresponding authors, and last/senior authors were 34%, 21%, and 18%, respectively. Note that, 17% of authors of clinical trial publications were females. Gender disparities in MM publications exist and are more obvious in the last/corresponding authorship. Efforts should be made to identify factors that contribute to these disparities and work to resolve them.

## 1 INTRODUCTION

Publications play an important role in advancing the field of oncology and are integral to helping authors gain credibility and advance their careers [1]. Therefore, identifying potential biases and disparities in the authorship of medical publications is crucial.

Previous studies have reported significant gender disparities and underrepresentation of women in medical peer‐reviewed publications which may result in job dissatisfaction and burnout [2, 3]. Women are less likely to progress in academia and earn full professorship ranks [4], be primary authors in major journals of original research [5], and hold leadership roles such as medical school deans and department chairs [7]. Although gender disparities have been studied in many different medical specialties, there is a paucity of data in the hematology/oncology field of research and specifically in multiple myeloma which has seen tremendous progress over the past decade [8, 9]. Therefore, we aimed to analyze whether gender disparities exist among authors of peer‐reviewed publications in the field of multiple myeloma (MM) in high‐impact hematology/oncology journals between the period of January 1, 2016, and December 31, 2020.

## 2 METHODS

Data on MM publications were collected through Medline/PubMed database search and filtered to only include articles from specific journals in the studied period. The following search items were used: “multiple myeloma”, “myeloma”, and “plasma cell dyscrasia”. Top journals in hematology/oncology, determined by an impact factor greater than 10, were included. Impact factor data was based on 2016 InCites Journal Citation Reports, an annual publication by Clarivate Analytics. We included all journals with an impact factor ≥10 with at least 10 MM‐related publications in the five years studied period. The selected journals include: the New England Journal of Medicine, Journal of Clinical Oncology, The Lancet, The Lancet Hematology, The Lancet Oncology, Blood, Nature Communications, Journal of Clinical Investigation, Nature Reviews of Clinical Oncology, Leukemia, and JAMA Oncology.

## 3 RESULTS

Our search yielded 750 publications which we further refined by excluding publications unrelated to multiple myeloma (*n* = 28) and specific publications types (news articles, narratives, and classical biographies) due to low research significance (*n* = 22). Of the remaining 700 publications, the following variables were collected: year of publication, field (clinical vs. basic), type of article (review, original investigation, clinical trial, case report, comment, editorial, letter, or meta‐analysis), number of authors, number of male authors, number of female authors, gender(s) of first author(s), gender(s) of last author(s), gender(s) of corresponding author(s), country of the first author, and country of the last author.

**FIGURE 1:**
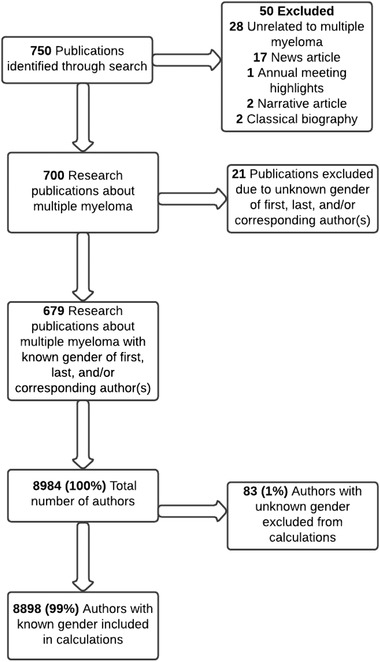
Flowchart for included studies

To determine the gender of the authors, we used Genderize, a validated database used in prior research to assess gender disparities in medical research publications [8, 10]. Genderize has also proven to be one of the most accurate among the most used gender determination tools in bibliometric studies [11]. If the gender could not be determined by the tool or did not meet our cut‐off of 0.9, we used ResearchGate and internet search engines to find photographs, profiles, or gender‐specific pronouns. First, last, or corresponding authors whose gender was not classified after a thorough search were marked as “unknown” and those publications (*n* = 21) were excluded from our analysis. Per inclusion criteria, a total of 679 publications with 8898 authorships were included in our analysis. [Figure 1]

**FIGURE 2:**
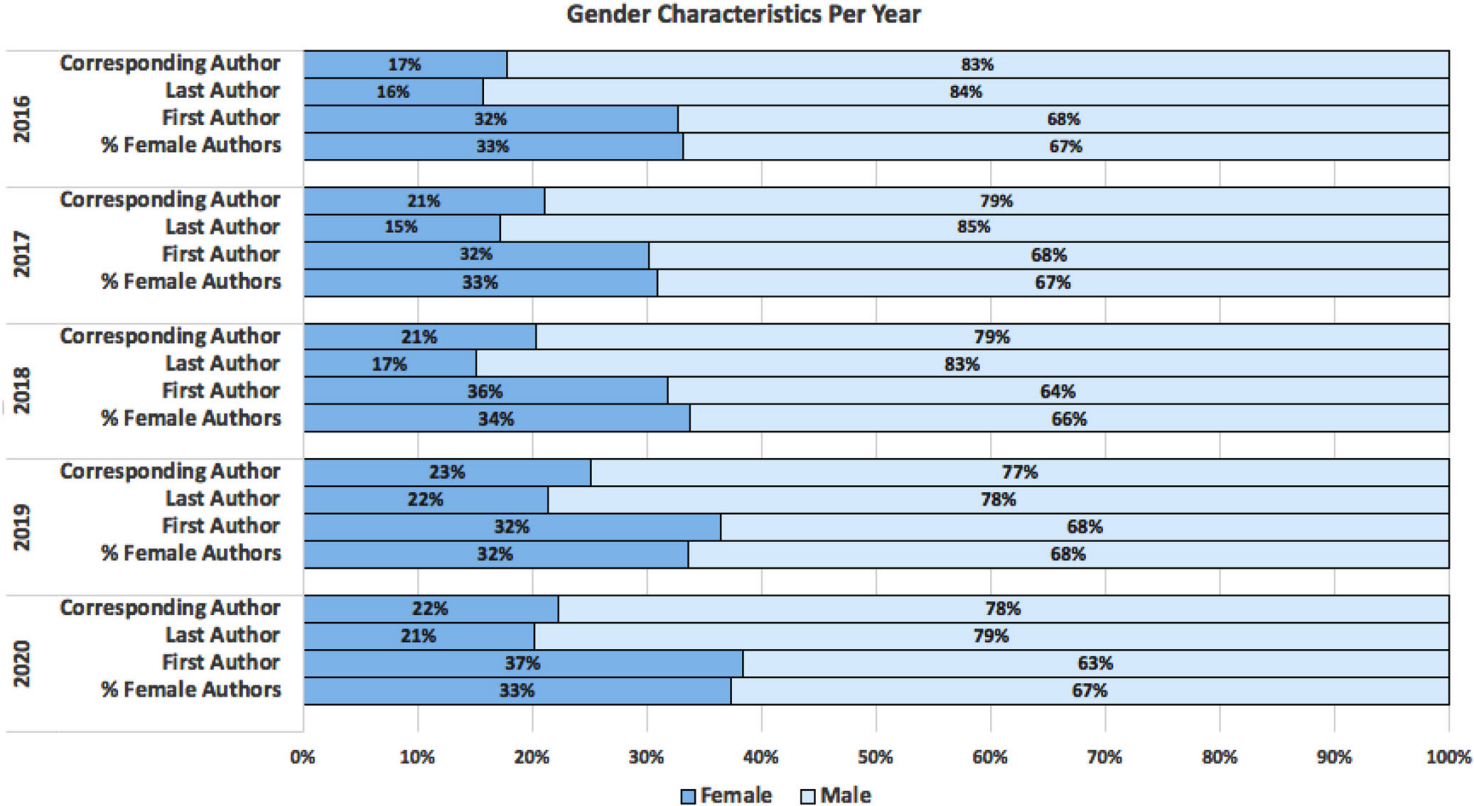
Gender characteristics per year

The average number of authors per publication was 13 (mean for females vs. males: 4.4 vs. 8.7). Females constituted around one third of total authors across all publications in the studied period. Females as first authors, corresponding authors and last/senior authors were 34%, 21%, and 18%, respectively. Figure 2 shows gender distribution among first authors, last authors, and corresponding authors per year. The number of female first authors, last authors, and corresponding authors were consistently lower than that of male first authors, last authors, and corresponding authors.

Original investigations and clinical trial publications constituted 61% of all publications in the studied period. Only 17% of authors of clinical trial publications were females. The proportion of females in original investigations publications was higher at 35%. The lower proportion of female authors was similar in basic vs. clinical research (32% vs. 36%, respectively). Data related to publication by country also revealed comparable percentages (30% females as first authors in the U.S. vs. 32% females as first authors in all other countries).

The proportion of female authorship was similar with no change in 2020 when compared to 2016 (both at 33%). There was a slight improvement in females as first authors (32%–37% from 2016–2020), last authors (16%–21% from 2016–2020), and corresponding authors (17%–22% from 2016–2020).

## 4 DISCUSSION

Our findings showed gender disparity in MM publications. Gender disparities were previously reported in other fields of medicine including in the hematology/oncology field [12, 13]. Females remain much less likely to publish and be first, last, or corresponding authors when compared to males. Gender disparities of female authorship in MM publications were especially prevalent among the proportion of the last authorship, which represents an important position in a manuscript denoting the senior/corresponding authorship in a publication. Our results are consistent with the low proportion of females as last/corresponding authors previously described in the field of general oncology [13].

Our analysis has some limitations. We only included publications of MM and we did not study the frequency of authors with multiple publications. Furthermore, the use of Genderize, an external database, to classify gender poses a risk of gender misclassification. Despite these limitations, our study provides a detailed analysis of all MM publications in medical journals focusing on the highest impact factor which has previously not been done.

In conclusion, gender disparities exist in MM peer‐review publications both in the US and other countries especially in the last/corresponding authorship. There is a slight improvement in the proportion of female authors, however, this is far from optimal given the growing proportion of female physicians in the medical field. Such disparities may contribute to increased burnout encountered by female physicians and may result in loss of interest in academic positions. Efforts should be made to identify factors that contribute to these disparities at all levels and implement strategies to support females and eliminate gender disparities in academia.

## FUNDING INFORMATION

The authors received no specific funding for this work.

## CONFLICT OF INTEREST

Aala Dweik, Hadeel Dweik, Meera Mohan, and Samer Al Hadidi report no relevant conflict of interest. Hira Mian receives advisory fees from Janssen, GSK, Celgene/BMS, Takeda, Sanofi, and research funding from Janssen. Hira Mian is the recipient of an early career research award from Hamilton Health Sciences.

## AUTHOR CONTRIBUTIONS

Samer Al Hadidi conceived the research idea. Samer Al Hadidi, Aala Dweik, and Hadeel Dweik performed the initial literature review. Aala Dweik, Hadeel Dweik, and Samer Al Hadidi collected data, performed the statistical analysis, and wrote the initial draft of the manuscript. Hira Mian, Carolina Schinke, and Meera Mohan provided critical input on the methodology and analysis. All authors reviewed and approved the final version of the manuscript.
